# Rotavirus A shedding and HBGA host genetic susceptibility in a birth community-cohort, Rio de Janeiro, Brazil, 2014–2018

**DOI:** 10.1038/s41598-020-64025-0

**Published:** 2020-04-24

**Authors:** Carina Pacheco Cantelli, Alvaro Jorge Velloso, Rosane Maria Santos de Assis, José Júnior Barros, Francisco Campello do Amaral Mello, Denise Cotrim da Cunha, Patricia Brasil, Johan Nordgren, Lennart Svensson, Marize Pereira Miagostovich, José Paulo Gagliardi Leite, Marcia Terezinha Baroni de Moraes

**Affiliations:** 1 Immunobiological Technology Institute/Bio-Manguinhos, Fiocruz, Avenida Brasil, 4365 Manguinhos, Rio de Janeiro Brazil; 2 Laboratory of Comparative and Environmental Virology, Oswaldo Cruz Institute, Fiocruz, Avenida Brasil, 4365 Manguinhos, Rio de Janeiro Brazil; 3 Laboratory of Molecular Virology, Oswaldo Cruz Institute, Fiocruz, Avenida Brasil, 4365 Manguinhos, Rio de Janeiro Brazil; 4 Laboratory of Viral Hepatitis, Oswaldo Cruz Institute, Fiocruz, Avenida Brasil, 4365 Manguinhos, Rio de Janeiro Brazil; 5 Sérgio Arouca National School of Public Health, Fiocruz, Avenida Brasil, 4365 Manguinhos, Rio de Janeiro Brazil; 6 Evandro Chagas National Institute of Infectious Diseases, Fiocruz, Avenida Brasil, 4365 Manguinhos, Rio de Janeiro Brazil; 7 Division of Molecular Virology, Department of Clinical and Experimental Medicine, Linköping University, 581 85 Linköping, Sweden

**Keywords:** Sequencing, Live attenuated vaccines, Gastrointestinal diseases

## Abstract

Recent studies have investigated whether the human histo-blood group antigen (HBGAs) could affect the effectiveness of the oral rotavirus vaccines, suggesting secretor positive individuals develop a more robust response. We investigated the Rotavirus A (RVA) shedding in association with the host susceptibility profile in children from a birth community-cohort in Rio de Janeiro, Brazil, from 2014 to 2018. A total of 132 children were followed-up between 0 to 11-month-old, stool samples were collected before/after the 1^st^/2^nd^ RV1 vaccination doses and saliva samples were collected during the study. RVA shedding was screened by RT-qPCR and G/P genotypes determined by multiplex RT-PCR and/or Sanger nucleotide sequencing. The sequencing indicated an F167L amino acid change in the RV1 VP8* P[8] in 20.5% of shedding follow-ups and these mutant subpopulations were quantified by pyrosequencing. The HBGA/secretor status was determined and 80.3% of the children were secretors. Twenty-one *FUT2* gene SNPs were identified and two new mutations were observed. The mutant F167L RV1 VP8* P[8] was detected significantly more in Le (a+b+) secretors (90.5%) compared to non-secretors and even to secretors Le (a−b+) (9.5%). The study highlights the probable association between RV1 shedding and HBGAs as a marker for evaluating vaccine strain host susceptibility.

## Introduction

After implementation of the rotavirus vaccines, Rotarix^®^ (RV1, GlaxoSmithKline Biologicals, Rixensart, Belgium) and RotaTeq® (RV5, Merck Inc., USA), in over 100 countries worldwide^1^, the burden of severe group A rotaviruses (RVA) diarrhea has decreased substantially, with reductions in hospitalizations and deaths in many countries, including Brazil^2–6^. However, RVA still are one of major causes of severe viral diarrhea in infants and young children <5-years-old worldwide^6,7^. Due to the zoonotic potential, variability of the RVA strains and host genetic factors, the surveillance of circulating RVA strains is necessary for evaluating and monitoring the effectiveness of the implemented immunization programs, mainly in low-income countries^8^. Currently, 36 G- and 51 P- genotypes have been described^9^ and globally, six G/P combinations are the most prevalent in humans: G1P[8], G2P[4], G3P[8], G9P[8], G4P[8] and G12P[8]^10^.

Studies involving the histo-blood group antigens (HBGA) have shown the importance of the VP8* domain (subunit of the VP4 protein of RVA); in the early stages of the pathogenesis of RVA infection^11–13^ as well as identifying RV1 G1P[8] shedding by nucleotide sequencing^14,15^.

HBGAs are complex glycans (type 1 H, Le^a^, Le^b^) present on cell surfaces and in biological fluids, such as blood, breast milk, saliva, and intestinal mucosa^16^. These glycans are catalyzed by glycosyltransferases, through sequential addition of monossaccharides to an initial precursor; these transferases are encoded by the *AB0*, *FUT2* (Secretor) and *FUT3* (Lewis) genes^17^. Secretor status is a host susceptibility factor in several infectious diseases, such as viral gastroenteritis by some RVA and noroviruses genotypes, and gastritis-ulcers by *H. pylori*^18^.

The HBGAs are highly polymorphic and differ across populations worldwide^17,19^. The epistatic interaction between *FUT2 (Se)* and *FUT3 (Le)* genes determines the Lewis phenotype^20^ and, principally, missense mutations in these genes interfere with the level of expression and activity of the α(1,2)-fucosyltransferase and α(1,3/1,4)-fucosyltransferase enzymes, determining ‘non-secretor’ and ‘Lewis negative’ Le (a−b−) phenotypes, respectively^21–23^. The HBGA genes’ mutations distributed worldwide as single nucleotide polymorphisms (SNPs) work as identity markers favoring certain conditions of susceptibility or resistance to infections or disorders.

In most studies on interaction between HBGAs and RVAs, the P[4] and P[8] VP8* genotypes preferentially infect the secretor and/or (Le^b^) individuals^24^; while non-secretor individuals (Le^a^) were less susceptible to these genotypes^25–27^. Recent studies have investigated whether the HBGA profile could contribute to the effectiveness of oral RVA vaccines^28–33^, suggesting secretor positive individuals develop a more robust response^28–30^.

This prospective study aimed to follow newborns up to <1-year of age in a low-income community-cohort in Manguinhos, Rio de Janeiro, Brazil, vaccinated with RV1 between November 2014 and November 2018, in order to assess the G1P[8] vaccine shedding in association to HBGA profile.

## Results

### Sampling of the children from Manguinhos community, Rio de Janeiro

The 132 children were followed for 16,212 child-days, ranging from 7–285 days, with 79% monitored up to at least 90 days. A total of 569 stool samples were collected and a median of five samples were obtained per child (no less than two samples/per child) and 132 saliva samples were obtained (1 sample/per child). Gender distribution was 50.8% (67) male and 49.2% (65) female.

### Rotavirus A shedding

A total of 19.2% (109/569) of stool specimens were positive for RVA by one-step reverse transcription-quantitative polymerase chain reaction (RT-qPCR), corresponding to 62.1% (82/132) of all children enrolled in this prospective study. By age range analysis, the highest RVA detection rate (39.3%, 92/234) was observed in children aged between 2 and 5 months (vaccination period) (Table 1). RVA acute diarrheic episodes (ADE) and non-ADE cases corresponded to 26.8% (11/41) and 18.5% (98/528) of stool samples respectively, and the G1P[8] was the most prevalent genotype (88.1%, 96/109). The G12P[8] (1.8%, 2/109), G3P[8] (0.9%, 1/109), and G3P[9] (0.9%, 1/109) genotypes were also detected. Four samples (3.7%) were G- not typed (G[NT]P[8]) and five samples (4.6%) were G- and P-not typed (G[NT]P[NT]).

**Table 1:** Stool samples collected from the 132 infants/children in acute diarrheic episodes (ADE) or non-ADE, rotavirus A (RVA) detection rate and G- and P-genotyping (NT = not typed) in the different age groups.

RV1 immunization period (age group)	n children (follow-up)	RVA detection rate (%)	RVA genotypes (ADE or non-ADE cases)	Secretor profile in RVA cases
Before the 1^st^ dose (<2 months)	127	11/304 (3.6)	5 G1P[8] and 3 RV1 G1P[8] (8 non-ADE)	8 *Se*
			G[NT]P[NT] (3 non-ADE)	3 *Se*
1^st^ and 2^nd^ doses (2–5 months)	125	92/234 (39.3)	6 G1P[8] and 78 RV1 G1P[8] (84 non-ADE)^a^	55 *Se*^b^ and 12 *se*^c^
			RV1 G1P[8] (2 ADE) and F167L RV1 G1P[8] (1 ADE)	2 *Se* and 1 *se*
			G12P[8] (1 ADE)	1 *Se*
			G3P[9] (1 ADE)	1 *Se*
			G[NT]P[NT] (2 non-ADE)	1 *Se* and 1 *se*
			G[NT]P[8] (1 non-ADE)	1 *se*
After the 2^nd^ dose (6–11 months)	24	6/31 (19.4)	RV1 G1P[8] (1 ADE) and G3P[8] (1 ADE)	1 *Se* (same child)
			G12P[8] (1 ADE)	1 *Se*
			G[NT]P8 (3 ADE)	3 *Se*
Total	132	109/569 (19.2)		

Distribution between RVA genotypes detected and the Secretor (*Se*: secretor, *se*: non-secretor) profile corresponding to 82 children followed. The birth community-cohort study was conducted between November 2014 and November 2018, in Rio de Janeiro, Brazil. ^a^In three children (5 G1P[8] cases), the secretor profile was considered inconclusive. ^b^Eight children with 2 RVA cases, ^c^four children with 2 RVA cases. F167L RV1 G1P[8] and RV1 G1P[8] and were characterized by Sanger sequencing, corresponding to the VP8* mutant gene for the F167L aa variation and the non-mutant, respectively. Cases of ADE with RV1 G1P[8] shedding are underlined.

Through nucleotide analysis of the VP8* gene by sanger sequencing, the G1P[8] strains were characterized as RV1 G1P[8] shedding in 88.5% of stool samples (85/96), with a range of 99.6–100% and 99.5–100% for nucleotide (nt) and amino acid (aa) similarity with RV1, respectively. Additionally, 33% (28/85) of the samples characterized as RV1-sourced G1P[8] were confirmed by Sanger sequencing of the VP7 (G1) and NSP4 genes presenting >99% of similarity (data not showed).

The RV1 G1P[8] in the analyzed strain was detected before the 1^st^ dose (3 non-vaccinated cases) and from day 1 to day 58 (median of 7 days) post-vaccination (1^st^ dose: 59 stool samples; 2^nd^ dose: 23 stool samples), with the Ct value (RT-qPCR) varying from 19.6 to 40 (median Ct value 35.6, range from 1.6 × 10^0^ to 2.4 × 10^5^ genome copies/mL).

ADE was detected in two RV1 vaccinated children, one 2 and one 6-month-old (1^st^ and 2^nd^ doses, respectively), and in Sanger sequencing of the VP8* gene from both children’s stool samples, RV1 G1P[8] shedding was detected. The RV1 G1P[8] recovered from the 2-month-old child’s stool presented a VP8* P[8] mutation.

In relation to the other non-G1P[8] genotypes detected in this cohort in acute diarrheic episodes (ADE), the G3P[9] genotype was observed 11 days after the 1^st^ dose of RV1 (RT-qPCR Ct value of 19) and G3P[8] was detected in one 6 month-old child (RT-qPCR Ct value of 32.5). The G12P[8] genotype was detected in one 4-month-old child (7 days after the RV1 2^nd^ dose, RT-qPCR Ct value of 39.1) and one 10-month-old child (RT-qPCR Ct value of 36.4).

### Detection of VP8* P[8] gene mutation

The F167L VP8* gene mutation in the RV1 G1P[8] characterized strains (F167L RV1 VP8* P[8]) was detected in 20.5% (27/132) of children monitored with shedding and was present in 23 children after the 1^st^ dose, 2 children after the 2^nd^ dose, 1 child before the 1^st^ dose (persisting after the 1^st^ dose, and 1 child before the 1^st^ vaccine dose; see Supplementary Table S1). The F167L RV1 VP8* P[8] was detected from day 5 to day 31 (median of 8 days, except for 2 cases detected before the 1^st^ dose), with a median RT-qPCR Ct value of 32.9. The RV1 G1P[8] shedding period (days post-vaccination) for children releasing these particles either with or without the F167L mutation was similar (median of 7 and 8 days).

Nucleotide analysis of the RV1 VP8* P[8] shedding by sanger sequencing showed a mutation at nt 499 T > C (1^st^ position of the codon, T/CTT) in 26 samples (30.6%, 26/85), besides the mutation at nt 501 T > A (3^rd^ position of the codon, T/CTA/T) in 2 samples (2.4%, 2/85), both positions resulting in the altered aa in position 167 in the VP4 protein (phenylalanine - Phe, substituted by leucine - Leu) (Table 2). Results obtained from pyrosequencing showed an allelic quantification of C/T for the 1^st^ position and T/A for the 3^rd^ position of the T/CTA/T codon, demonstrating a mixture of virus subpopulations containing Phe (TTT) and Leu (CTT, TTA or CTA) aa in RV1 G1P[8] characterized shedding. The stool samples varied from 1 to 95.5% (TTT) and 4.5% to 99% (CTT) in the 1^st^ position (see Supplementary Fig. S1 and Table 1), 14 to 4% (CTA) and 86 to 96% (CTT), and 68 to 89% (TTA) and 32 to 11% (TTT) in the 3^rd^ position. Besides that, two other aa substitutions were detected in the F167L RV1 VP8* P[8] shedding (in two stool samples): one (Y80H) in a conserved region and the other (N87S) in a hypervariable region (antigenic site 8–4). Furthermore, one silent mutation was detected in the nt position 219 A > G (aa 73 T), in other sample.

**Table 2:** Nucleotide (nt) substitutions and corresponding 167 amino acid substitution (aa, Leu: leucine or Phe: phenylalanine) in the VP8* gene in different rotavirus A (RVA) strains: G1P[8] *Wa-like*, vaccine prototype, RV1 vaccine and RV1 G1P[8] shedding detected in the Manguinhos community-cohort, Rio de Janeiro, Brazil, from November 2014 to November 2018.

Rotavirus A P[8] strains	amino acid position 167			
	Codon			
	1^st^ (nt 499)	2^nd^ (nt 500)	3^rd^ (nt 501)	aa
*Wa-like* (GenBank accession number: JX406750)	T	T	G	Leu
Prototype P1A[8] unpassaged 89–12	T	T	G	Leu
RV1 vaccine	T	T	T	Phe
RV1 shedding (this study)	T	T	T	Phe
	C	T	T	Leu
	T/C	T	A/T	Leu

The RV1 vaccine was used for VP8* Sanger sequencing and corresponds to the batch GlaxoSmithKline Biologicals Rixensart – Belgium, Human Rotavirus Live Attenuated RIX4414 strain – 1 dose, AROLB385A - Fab: February/2015 – Expiration date: January/2017.

### HBGA phenotyping and FUT2 genotyping

Regarding the secretor status, 80.3% (106/132) of the children were classified as secretors and 15.9% (21/132) were non-secretors. Secretor status definition for 3.8% (5/132) of children was inconclusive. The Lewis phenotypes detected were: 59.8% (79/132) Le (a+b+), 15.9% (21/132) Le (a − b − ), 13.6% (18/132) Le (a − b + ), and 10.6% (14/132) Le (a+b−).

*FUT2* genotyping was performed for 78 children with Le (a+b+) secretor and 21 with non-secretor status. Twenty-one SNPs were determined in 82.1% (64/78) of the Le (a+b+) secretor children and 17.9% (14/78) of them did not present any SNP. Twenty-eight genotypes were identified in Le (a+b+) secretors children (see Supplementary Table S2) and the most frequent genotype was *Se* ^*171A>G, 216C>T, 357T>C*, 428*G>A, 739G>A, 960A>G, 1009A>G, 1011T>C*^ (35.9%, 28/78). The rs281377 SNP (357 C > T) was detected in high frequency (80.7%, 63/78) and two new mutations were detected in the Le (a+b+) secretor phenotype (107 T > A and 257 C > T). Twelve SNPs were detected at a low frequency: rs1800021 (40 A > G), rs138507381 (212 T > C), rs200157007 (302 C > T), rs28362836 (315 C > A), rs1800026 (375 A > G), rs1800027 (480 C > T), rs1800025 (481 G > A), rs148371614 (544 G > A), rs142741127 (771 G > A), rs141630650 (855 A > C), rs916106939 (880 T > C), and rs144809245 (969 C > T). The rs1047781 SNP (385 A > T) was not detected in any children phenotyped as Le (a+b+) in this cohort. Twelve (58.3%, 14/21)

children classified as non-secretor presented the following homozygous genotype: *se* ^*171A>G, 216C>T, 357T>C, 428G>A, 739G>A, 960A>G, 1009A>G, 1011T>C*^ (see Supplementary Table S3). In addition, one non-secretor child presented a new mutation (257 C > A) and the SNPs rs28362836 (315 C > T), rs138954645 (542 C > T), rs148371614 (544 G > A) and rs142741127 (771 G > A) were identified at a low frequency in three children, and were considered heterozygous at these positions (see Supplementary Table S3). One non-secretor child did not present any SNP in the *FUT2* gene.

### Rotavirus A shedding and host susceptibility

Six genotypes in the *FUT2* gene were identified in 69.6% (16/23) of the Le (a+b+) secretor children with F167L RV1 VP8* P[8] shedding, and rs281377 (357 C > T) was present in 100% (16/16) of them. F167L RV1 VP8* P[8] shedding was more commonly detected in children with secretor status (*p* = 0.0433), and a Le (a+b+) phenotype (*p* = 0.0354) (Tables 3 and 4).

**Table 3:** Association between Secretor and Lewis profile and G1P[8] RV1 shedding (n = 61 children), without and with a F167L mutation, in the birth community-cohort, Rio de Janeiro, Brazil, from 2014 to 2018^a^.

Secretor/Lewis profile	RV1 G1P[8] shedding^a^		F167L RV1 G1P[8] shedding^b^		*p* value^c,d^
	n = 40	(%)	n = 21	(%)	
Secretor	30	(75)	21	(100)	0.0433
Non-secretor	9	(22.5)	0	(0)	
Indetermined	1	(2.5)	0	(0)	
Le(a+b−)	6	(15)	0	(0)	0.0354
Le(a−b+)	5	(12.5)	2	(9.5)	
Le(a+b+)	23	(57.5)	19	(90.5)	
Le(a−b−)	6	(15)	0	(0)	

The immunization schedule of the children with RV1 G1P[8] shedding: 28 after 1^st^ dose, 12 after 2^nd^ dose and 1 non-vaccinated child. ^b^All children were vaccinated with the 1^st^ dose, except one (non-vaccinated). ^c^Twelve children (with 2 RVA positive results each) were removed of the statistic analysis. ^d^*P* values were determined by Chi-square test.

**Table 4:** Detection of the RV1 G1P[8] shedding, with or without a F167L mutation, in children phenotyped as Le (a+b+) secretors (n = 47) in the birth community-cohort, Rio de Janeiro, Brazil, from 2014 to 2018.

Code Child	Immunization schedule	Days post-vaccine	RVA RT-qPCR Ct value	VP8* gene(Sanger sequencing)
004	3	NA	38,3	F167L RV1 G1P[8]
033	1	8	38,5	F167L RV1 G1P[8]
037	1	8	39,5	RV1 G1P[8]
043	1	5	34,1	F167L RV1 G1P[8]
053	1	5	39,7	RV1 G1P[8]
057	1	8	34,9	F167L RV1 G1P[8]
071	2	5	33,7	RV1 G1P[8]
072	2	5	30,3	RV1 G1P[8]
090	1	10	33,1	F167L RV1 G1P[8]
094	1	9	31,6	F167L RV1 G1P[8]
096	1	6	36,6	RV1 G1P[8]
097	1	8	37,2	RV1 G1P[8]
099	1	16	38,1	RV1 G1P[8]
101	1	8	34,1	F167L RV1 G1P[8]
113	1	7	28,4	F167L RV1 G1P[8]
122	1	7	35,8	RV1 G1P[8]
	2	?	37,5	F167L RV1 G1P[8]
142	1	17	36,7	F167L RV1 G1P[8]
144	1	12	26,3	F167L RV1 G1P[8]
160	1	12	33,9	F167L RV1 G1P[8]
164	2	7	36,7	RV1 G1P[8]
173	2	9	35,4	RV1 G1P[8]
204	1	8	38,0	RV1 G1P[8]
211	1	8	32,8	F167L RV1 G1P[8]
225	1	7	23,3	F167L RV1 G1P[8]
226	2	8	37,7	RV1 G1P[8]
233	1	8	37,2	RV1 G1P[8]
234	1	5	24,5	F167L RV1 G1P[8]
	2	9	35,4	RV1 G1P[8]
235	1	8	21,9	F167L RV1 G1P[8]
236	1	8	19,8	F167L RV1 G1P[8]
240	1	9	39,6	F167L RV1 G1P[8]
244	1	8	19,6	F167L RV1 G1P[8]
245	1	6	21,6	RV1 G1P[8]
249	2	5	34,5	RV1 G1P[8]
250	1	6	22,6	RV1 G1P[8]
253	1	22	30,0	F167L RV1 G1P[8]
259	1	6	26,5	F167L RV1 G1P[8]
	2	7	30,7	RV1 G1P[8]
261	1	7	22,7	RV1 G1P[8]
	2	24	36,1	RV1 G1P[8]
264	1	5	27,1	RV1 G1P[8]
265	2	58	38,6	RV1 G1P[8]
266	2	10	33,7	RV1 G1P[8]
270	3	NA	21,0	F167L RV1 G1P[8]
	1	31	32,9	F167L RV1 G1P[8]
275	1	6	20,3	RV1 G1P[8]
281	1	6	19,9	F167L RV1 G1P[8]
282	1	3	32,0	RV1 G1P[8]
284	1	4	26,6	RV1 G1P[8]
292	1	4	22,9	RV1 G1P[8]
293	1	5	35,9	RV1 G1P[8]

G1P[8] RVA samples were firstly genotyped by G/P multiplex RT-PCR. The VP8* gene was analyzed by sanger nucleotide sequencing. Information about these derived vaccine samples are summarized: the immunization schedule (1: 1^st^ dose, 2: 2^nd^ dose, 3: non-vaccinated), number of days of vaccine shedding (NA: not applicable,?: no information) and Rotavirus (RVA) RT-qPCR Ct value.

Other RVA genotypes isolated in ADE in this cohort were detected in the secretor children, these being G12P[8] in Le (a−b+) and Le (a+b+) and G3P[8] in Le (a+b+). The G3P[9] RVA was isolated from a child both Le (a−b−) and positive for fucose detection. A P[8] genotype, untypable for G[NT], was detected in three Le (a+b+) secretor children and in one secretor Le (a−b+) child. Untypable (G[NT]P[NT]) RVA cases were detected in Le (a+b+) secretors (four children) and in one non-secretor Le (a+b−) child.

## Discussion

This prospective study focused mainly on assessing the RVA shedding in association to the HBGA profile in a birth community-cohort in Rio de Janeiro, Brazil. Through monitoring the RV1 G1P[8] shedding, it was possible to detect the F167L RV1 VP8* P[8] from stool samples of children from prior to the 1^st^ dose and during the RV1 immunization period. The evaluation of these children’s host susceptibility profile showed that secretor as well as Lewis b positive children were significantly more likely to shed RV1 G1P[8] with occurrence of the F167L VP8* mutation.

Via VP8* P[8] Sanger sequencing, 88.5% of the G1P[8] RVA detected were identified as RV1, mainly after the 1^st^ dose (96.5%), at 2 months of age, similar to previously documented in clinical trials^34–36^. The G1P[8] genotype was detected in eight non-vaccinated children (before the 1^st^ dose) and due to the low viral load, only three cases could be sequenced and determined as RV1 G1P[8]. Despite the VP8* P[8] Sanger sequencing having been used to differ between RV1 and non-RV1 shedding^14,15^, we should strictly consider the possibility of the RV1 G1P[8] shedding here characterized containing a RVA constellation differing from the source RV1. Therefore, this study shows the importance of characterizing the RV1 via the RVA constellation method before a vaccine batch can be used to produce RV1 doses.

The horizontal transmission of vaccine viruses has been previously demonstrated between vaccinated and unvaccinated infants or other close contacts^35,36^. RV1 G1P[8] shedding was reported in two diarrhea cases after the 1^st^ dose, with the F167L mutation being detected in one of them. In this study we observed a low diarrhea incidence, mainly in the first 4 months of age, which could be related to RV1 protection and the effect of exclusive breastfeeding (and transferred maternal antibodies) in these first months of life^37^.

In the few diarrhea cases detected in this study, a rare G3P[9] was identified in one 2-month-old child after the 1^st^ dose, with the VP8* P[9] gene exhibiting 97% nucleotide similarity to an *AU-1-like* Brazilian strain (KJ820906) collected from an inpatient 2-year-old, indicating a possible feline/canine-to-human interspecies transmission^38^. The effectiveness of the RVA vaccines against genogroups 1 and 2 (*Wa-like* and *DS-1-like* strains, respectively) has been proven, but still remains unclear against the genogroup 3 (*AU-1-like*)^39,40^. As to this rare genotype detected in Manguinhos, further studies are being conducted to determine the complete genomic constellation.

Through our molecular characterization of the RV1 G1P[8] shedding, it was possible to observe a high frequency of the mutation in the 167 position of the VP8* P[8] gene. In our study, only the VP8* domain was analyzed because it is directly related to interactions with HBGAs. In the previous studies with the G1P1A[8] rotavirus vaccine candidate 89‐12, the precursor to RV1, Ward *et al*.^41^ identified five aa changes in the VP4 gene (G51D and L167F in VP8* domain; D331Y, D385Y and N695I in VP5* domain) that occurred during vaccine attenuation. It was proposed that these alterations could reduce cross neutralizing antibody responses. Indeed, according to Gozalbo-Rovira *et al*.^42^, the 167 position is placed at the bottom of the sugar-binding pocket, and Phe residue contained in RV1 G1P[8] could influence the interaction between the vaccine strain and the sugar of the cellular receptor, due to hydrophobic residue decreasing the binding affinity. 32.9% of the RV1 G1P[8] shedding samples detected in this birth-cohort presented the mutation in the 167 position, with a mixture of virus subpopulations of both amino acids, Leu (CTT, TTA, CTA) and Phe (TTT), in different percentages. We could hypothesize that this suggest an initial process of reversion of attenuation or a selective pressure, through the one and/or two alteration(s) in this codon (positions nt 499, 501), favoring the highest amount of circulation of strains containing the Leu residue, which has a greater avidity for the HBGA’s binding site in the secretor profile. Zeller *et al*.^43^ also reported the mutation in position 167 in the 3^rd^ position of the codon (nt 501), in one vaccine derived strain (BE00048) detected in Belgium, in 2009.

Positive secretor status was the most prevalent (80.3%) in this community-cohort, and the Le (a+b+) profile (59.8%) frequency was similar to that observed in younger children from the Amazon (58.8%) as presented in our previous study^44^. The rs1047781 (385 A > T) SNP, responsible for the weak genotype in the Asian population, was not detected in Le (a+b+) children in the Manguinhos community, Rio de Janeiro state. The rs281377 (357 C > T), a synonymous mutation, was the most frequent SNP detected in this Le (a+b+) secretor profile, and this SNP has been detected in the Brazilian population^44,45^. According to Ferrer-Admetlla *et al*.^19^, the rs281377 (357 C > T) SNP has been detected in a worldwide distribution profile in the natural population, and the *se*^*357/385*^ haplotype is frequently detected in the Asian population. We could explain the high frequency of the Le (a+b+) phenotype detected in children in Rio de Janeiro, Brazil under an evolutionary view that, at some point in the molecular clock, the *se*^*357/385*^ haplotype had diverged and separated from the rs1047781 (385 A > T) SNP. However, epistatic interactions between rs281377 (357 C > T) and other SNPs could affect the expression of the *FUT2* gene. The failure in detecting the rs1047781 (385 A > T) SNP, which has been attributed as responsible for the weak secretor phenotype detected in Le (a+b+), in the children in the Manguinhos community in the state of Rio de Janeiro could be explained by the presence of new SNPs in conjunction with rs281377 (357 C > T). Further studies encompassing larger populations, including Le (a − b + ) secretors will be needed to evaluate whether particular SNPs in *FUT2* gene can explain the phenotypes Le (a+b+) *vs* Le (a − b + ) (or similar).

Our study identified SNPs in the *FUT2* gene in children from the Manguinhos community, in Rio de Janeiro as rs1800021 (40 A > G), rs48703160 (171 A > G), rs681343 (216 C > T), rs281377 (357 T > C), rs601338 (428 G > A), rs602662 (739 G > A) and rs485186 (960 A > G), also reported by Vicentini *et al*.^45^ in children from a Quilombola community (black population, slave descendants) in Espírito Santo (borders with the state of Rio de Janeiro, both states in Southeastern Brazil).

The Brazilian population has a high ethnical diversity and through this study in the Manguinhos children community-cohort, it was possible identify two new mutations: 107 T > A and 257 C > T/A and other SNPs, at a low frequency, not yet reported in Brazil: rs138507381 (212 T > C), rs200157007 (302 C > T), rs28362836 (315 C > A/T), rs1800026 (375 A > G), rs1800025 (481 G > A), rs138954645 (542 C > T), rs148371614 (544 G > A), rs142741127 (771 G > A), rs141630650 (855 A > C), rs916106939 (880 T > C), and rs144809245 (969 C > T).

Le (a+b+) phenotype is very common in infants and children under 2 years-old, usually being a transient status in most children^46^. In the infancy period, the Lewis phenotypes have not yet fully matured^47^, due to the activity of the fucosyltransferases (*FUT2* and *FUT3* genes) not yet reaching the normal levels (enzyme activity of *Le* > *Se*)^20^.

In relation to the non-secretor phenotype, a unique child (0Le (a−b−)) did not present any SNP in the *FUT2* gene, i.e., not showing the nonfunctional allele *se*^*428*^ (rs601338)^21^. This child shed the RV1 G1P[8] strain virus in both doses (6 days post-1^st^ dose; 7 days post-2^nd^ dose); however, only in the 2^nd^ dose was the F167L mutation detected, with mixed virus subpopulations of 63.5% Phe/36.5% Leu. Possibly, other genetic factors could be related to this profile and vaccinal response.

The P[9] RVA genotype interacts with A-type HBGA antigen^48^, and the AB0 enzyme assay was performed in a saliva sample from the unique child that was infected with this genotype (G3P[9]) in this cohort, presenting positivity for type A antigen (ALe (a−b−) secretor) (data not shown), thus in line with the proposed susceptibility of A to genotype P[9].

The Manguinhos cohort showed a high prevalence of secretors, being a common profile in Latin America^30,31,44,45,49^. In this study, we were able to investigate RVA shedding and a common mutation was identified in many samples characterized as RV1 according to VP8* Sanger sequencing criteria. This mutational event suggests that the vaccine could be replicating and therefore a more robust immune response may be produced in these infants. In summary, our study demonstrated that Le (a+b+) secretors can influence vaccine replication.

This study highlights the detection of the new mutations in the *FUT2* gene detected in the community of Manguinhos, Rio de Janeiro, Brazil and the association between vaccine strain shedding and HBGAs as a marker for evaluating vaccine efficiency. The results in this study indicate the importance of the full molecular monitoring of the RV1 vaccine virus stool shedding, mainly if the F167L VP8* mutation is frequent in other populations, and how the host susceptibility profile can influence this viral selective pressure, besides the questions about circulation and fluctuations of the emergent RVA genotypes and ensuring vaccine efficacy in a post-RVA vaccine era.

## Material and methods

### Study design, clinical specimens and Ethics Statement

This is a prospective cohort study that aims to perform acute gastroenteritis surveillance in the Manguinhos community, Rio de Janeiro, Brazil, which has one of the lowest Human Development Indexes (HDI) of the municipality^50^. This project has been approved by the Ethics Committee of the Evandro Chagas National Institute of Infectious Diseases (CEP 688.566/14). Written informed consent was obtained from each child’s parent or legal guardian. The RV1 vaccine was the one received by all vaccinated newborn infants enrolled in this study (2-dose series at ages 2 and 4 months and with a minimum interval between doses of 4 weeks). The vaccination card of each child was verified. A vaccination inquiry was performed at Germano Sinval Faria Health Center (GSFHC) by a pediatrician who accompanied this study, questioning each child’s parent or guardians as to vaccination for RVA (double-checking).

A flow diagram including the participants and the specific steps of methods applied in this study are presented in Supplementary Fig. S2. All clinical specimens were sent to the Regional Rotavirus Reference Laboratory/Laboratory of Comparative and Environmental Virology (RRRL-LCEV) and kept at −20 °C, until the moment of processing and further analysis. The methods were performed in accordance with guidelines and regulations.

### RNA extraction from fecal specimens and viral RVA detection

Viral RNA was extracted from clarified stool specimens (10–20% w/v) using an automatic RNA extraction procedure according to the manufacturer’s instructions (QIAcube^®^ Automated System and QIAamp^®^ Viral RNA Mini kit; Qiagen, CA, USA). RVA detection was performed using RT-qPCR on an Applied Biosystems® 7500 Real-Time PCR System (Applied Biosystems, Foster City, CA, USA) as previously described^51^. The RVA RT-qPCR result was considered positive if the cycle threshold (Ct) value was ≤ 40.

### G- and P- RVA genotyping

Semi-nested multiplex reverse transcription-polymerase chain reaction (RT-PCR) was performed for G- and P- RVA genotyping using SuperScript® III One-Step RT-PCR System with Platinum® Taq DNA Polymerase High Fidelity (Invitrogen) and 9con1L/VP7deg (VP7) and 4con3/4con2 (VP8*) primers to generate 904-base-pair (bp) and 876-bp fragments, respectively. These amplicons were used as a template in a second round of amplification with genotype-specific primers as described on WHO/IVB/08.17^52^. In parallel with the amplicons obtained from child stool samples, the batch named RV1 vaccine identified as GlaxoSmithKline Biologicals Rixensart – Belgium, Human Rotavirus Live Attenuated RIX4414 strain – 1 dose, AROLB385A - Fab: February/2015 – Expiration date: January/2017, was used for RNA extraction and VP8* gene amplification using the same 4con3/4con2 (VP8*) primers cited above.

### VP8* RVA molecular characterization

Products of one-step reverse transcription-polymerase chain reaction (RT-PCR) using 4con3/4con2 (876-pb) were purified using Wizard® SV Gel and a PCR Clean-Up System kit (Promega, Madison, USA) following the manufacturer’s instructions. The purified amplicons of the VP8* gene of RVA were analyzed by Sanger sequencing using a BigDye® Terminator v3.1 Cycle Sequencing Kit and the ABI Prism 3730 or 3500 Genetic Analyser^®^ (Applied Biosystems, Foster City, CA, USA). Chromatograms were analyzed and nt sequences (consensus) were edited using the BioEdit 7.2.1 Sequence Alignment Editor^53^ and nt similarity was assessed using the Basic Local Alignment Search Tool (https://blast.ncbi.nlm.nih.gov/Blast.cgi). RVA genotypes were assigned using the RotaC^2.0^ automated genotyping tool for Group A rotaviruses (https://rotac.regatools.be/)^54^. Deduced aa sequences of VP8* (G1P[8]) were aligned and compared with the RV1 (JX943612), RV1 vaccine and G1P[8] *Wa-like* (JX406750) strains, and nt and aa similarities values between RV1 and G1P[8] strains were verified using the BioEdit 7.2.1 Sequence Alignment Editor^53^. The representative gene sequences of VP8* RVA obtained in the current study were submitted to GenBank under the accession numbers MN366044-MN366074. Pyrosequencing for mutation analysis of RV1 VP8* P[8] shedding A pyrosequencing assay was performed using PyroMark Q96 ID (QIAGEN Valencia, CA, USA). The following primers were designed to amplify a 203-bp fragment of the VP8* P[8] subdomain containing the target (cytosine or thymine/nt 499, and adenine or thymine/nt 501): forward (VP8*RV1F) 5′-AGCAATTTAATGTGAGTAACGA-3′ (nt 346–368) and reverse (VP8*RV1R) 5′-BIOTIN-AAATTTGCAGTACTTGAACTGTCA-3′ (nt 548–525) using PyroMark® Assay Design 2.0 software (QIAGEN Valencia, CA, USA). The forward (VP8*RV1-S1) (5′-TGATACCAGACTTGTAGGA-3′; nt 447–465) was designed for the pyrosequencing assay. The biotinylated fragment (203-pb) was amplified using SuperScript® III One-Step RT-PCR System with Platinum® Taq DNA Polymerase (Invitrogen). Initially, the samples were denatured with primers at 97 °C/7 min. Next, the master mix was added in each tube, and incubated under the following thermocycling conditions: cDNA synthesis and pre-denaturation at 50 °C/30 min, denaturation at 94 °C/10 min, then 40 cycles of 94 °C/15 s, 53 °C/30 s and 68 °C/1 min, followed by a final elongation step (68 °C/5 min). Each sample and controls (positive and negative) were tested in duplicate and the quality of the RT-PCR products were checked by agarose gel electrophoresis. Biotinylated amplicons were hybridized to streptavidin-coated beads and purified using the PyroMark Q96 Vacuum Prep Workstation (Qiagen, Valencia, CA, USA) according to the manufacturer’s instructions. Pyrosequencing reactions were performed using the PyroMark Gold Q96 SQA Reagents in the PyroMark Q96 ID (QIAGEN) following the manufacturer’s instructions, and the analysis of the peaks was performed using PyroMark ID software. The allelic quantification results (%) were calculated by the duplicate media. The primers designed for this analysis and thermocycling conditions were previously tested and validated. As a positive control, a fragment of the VP8* P[8] region (encoding aa 7–257) of the RV1 stool shedding was isolated. The fragment without the mutation (nt 499/T) was ligated into the pCR^®^4-TOPO^®^ vector using the TOPO TA Cloning® Kit for Sequencing (Invitrogen, Life Technologies, UK) and then transformed into competent *Escherichia coli* Top 10 (Invitrogen, Life Technologies, UK) following the classical methodologies previous described^55^.

### HBGA phenotyping and secretor status in saliva

The saliva samples were collected using sterile cotton-swabs (CHEMBIO®, Medford, NY, USA). Two enzyme immunoassays (EIA) were performed, as described previously^44,56^, to detect AB0 (H) histo-blood groups and Lewis phenotypes, and specifically define the secretor status of Le (a−b−) saliva samples (using *Ulex europaeus* lectin).

### DNA extraction from saliva and FUT2 genotyping

Genomic DNA recovered from epithelial cells was extracted from each saliva sample collected from children phenotyped as Le (a+b+) secretor and non-secretor Le (a+b−) or Le (a−b−) and were used in the touchdown PCR for the genotyping of SNPs in the *FUT2* gene, by Sanger sequencing as previously described^44,57^.

### Statistical analysis

Statistical analysis was performed using GraphPad Prism 8 v.8.2.0 (GraphPad Software, San Diego, CA, USA). Detection of the G1P[8] RVA genotype (RV1 stool shedding) in association with the host susceptibility profile was investigated, when appropriate, through the Chi-square or Fisher’s exact test, with a significance level of 5.0%.

## Supplementary information

Supplementary File.
