# Supplementary material for: Rotavirus A shedding and HBGA host genetic susceptibility in a birth community-cohort, Rio de Janeiro, Brazil, 2014–2018
Source: Sci Rep. 2020 Apr 24;10:6965. doi: 10.1038/s41598-020-64025-0 (PMC7181595; doi:10.1038/s41598-020-64025-0)
Supplement: Supplementary file 1 — Supplementary File. [file 41598_2020_64025_MOESM1_ESM.docx]

**Rotavirus A shedding and HBGA host genetic susceptibility in a birth community-cohort, Rio de Janeiro, Brazil, 2014-2018.**

Carina Pacheco Cantelli*^1,2^ · Alvaro Jorge Velloso^1^ · Rosane Maria Santos de Assis^2^ · José Júnior Barros^3^ · Francisco Campello do Amaral Mello^4^ · Denise Cotrim da Cunha^5^ · Patricia Brasil^6^ · Johan Nordgren^7^ · Lennart Svensson^7^ · Marize Pereira Miagostovich^2^ · José Paulo Gagliardi Leite^+2^ & Marcia Terezinha Baroni de Moraes^+2^

^+^ These authors contributed equally to this work.

^1^Immunobiological Technology Institute/ Bio-Manguinhos, Fiocruz, Avenida Brasil, 4365,

Manguinhos, Rio de Janeiro, Brazil.

^2^Laboratory of Comparative and Environmental Virology, Oswaldo Cruz Institute, Fiocruz, Avenida Brasil, 4365, Manguinhos, Rio de Janeiro, Brazil.

^3^Laboratory of Molecular Virology, Oswaldo Cruz Institute, Fiocruz, Avenida Brasil, 4365, Manguinhos, Rio de Janeiro, Brazil.

^4^Laboratory of Viral Hepatitis, Oswaldo Cruz Institute, Fiocruz, Avenida Brasil, 4365, Manguinhos, Rio de Janeiro, Brazil.

^5^Sérgio Arouca National School of Public Health, Fiocruz, Avenida Brasil, 4365, Manguinhos, Rio de Janeiro, Brazil.

^6^Evandro Chagas National Institute of Infectious Diseases, Fiocruz, Avenida Brasil, 4365, Manguinhos, Rio de Janeiro, Brazil.

^7^Division of Molecular Virology, Department of Clinical and Experimental Medicine, Linköping University, 581 85 Linköping, Sweden.

^*^Corresponding author: Carina Pacheco Cantelli - ORCID: 0000-0002-9171-5022

Laboratory of Comparative and Environmental Virology, Oswaldo Cruz Institute – Fiocruz

Av. Brasil, 4365 – Pav. Hélio & Peggy Pereira, 21040-360 - Rio de Janeiro – RJ - Brasil

Phone: 55 21 - 2562-1817 / 2562-1851

email: carina.cantelli@gmail.com

**Supplementary information**


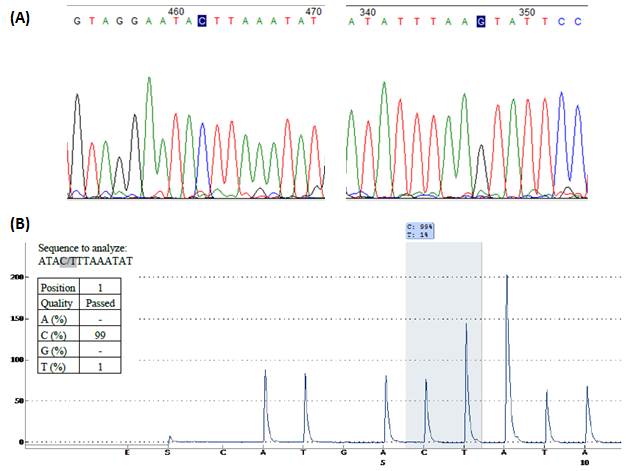

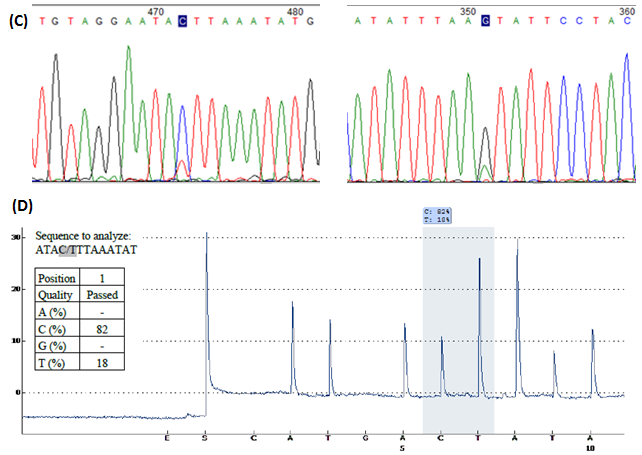


Figure S1: Chromatograms (Forward and Reverse, Sanger Nucleotide Sequencing, A and C) and Pyrograms (Pyrosequencing Method, B and D). Pyrosequencing showed a sensitivity of 1% in a mixture sample (F167L) in RV1 G1P[8] shedding (nt 499, 1% TTT, Phenylalanine and 99% CTT, Leucine).


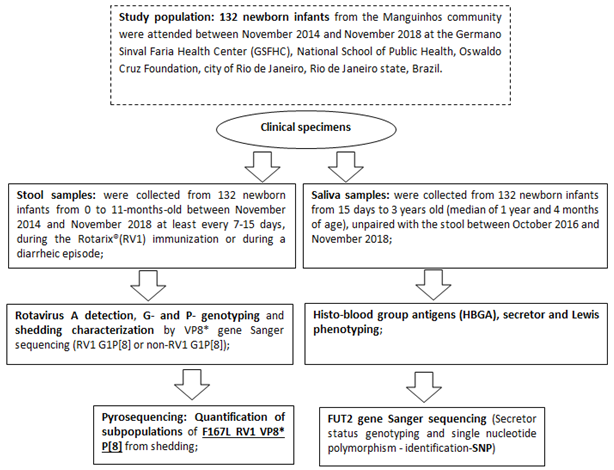


Figure S2: Flow diagram of the birth cohort-community study showing the population participants and laboratory tests.

| Child code | Secretor status | Lewis  status | Immunization schedule | Days post-vaccination | RVA RT-qPCR Ct value | Sanger Sequencing | Pyrosequencing | |
| --- | --- | --- | --- | --- | --- | --- | --- | --- |
|  |  |  |  |  |  | peak nt 499 | % TTT (Phe) | % CTT (Leu) |
| 004 | *Se* | Le (a+b+) | 3 | NA | 38.3 | T>C | 73.5 | 26.5 |
| 101 | *Se* | Le (a+b+) | 1 | 8 | 34.1 | C>T | 13 | 87 |
| 033 | *Se* | Le (a+b+) | 1 | 8 | 38.5 | C>T | 17 | 83 |
| 043 | *Se* | Le (a+b+) | 1 | 5 | 34.1 | C>T | 15.5 | 84.5 |
| 057 | *Se* | Le (a+b+) | 1 | 8 | 34.9 | C>T | 9 | 91 |
| 079 | *Se* | Le (a-b+) | 1 | 8 | 31 | T>C | 63.5 | 36.5 |
| 113 | *Se* | Le (a+b+) | 1 | 7 | 28.4 | C>T | 29 | 71 |
| 090 | *Se* | Le (a+b+) | 1 | 10 | 33.1 | T>C | 57 | 43 |
| 103 | *se* | Le (a-b-) | 2 | 7 | 36.3 | T>C | 63.5 | 36.5 |
| 094 | *Se* | Le (a+b+) | 1 | 9 | 31.6 | C | 2.5 | 97.5 |
| 144 | *Se* | Le (a+b+) | 1 | 12 | 26.6 | C | 2 | 98 |
| 122 | *Se* | Le (a+b+) | 2 | ? | 37.5 | C | 1 | 99 |
| 142 | *Se* | Le (a+b+) | 1 | 17 | 36.7 | C | 11.5 | 88.5 |
| 158 | *Se* | Le (a-b+) | 1 | 7 | 33.3 | C>T | 10 | 90 |
| 211 | *Se* | Le (a+b+) | 1 | 8 | 32.8 | C | 1.5 | 98.5 |
| 160 | *Se* | Le (a+b+) | 1 | 12 | 34 | T>C | 60.5 | 39.5 |
| 236 | *Se* | Le (a+b+) | 1 | 8 | 19.8 | T>C | 55.5 | 44.5 |
| 225 | *Se* | Le (a+b+) | 1 | 7 | 39.6 | C | 32.5 | 67.5 |
| 234 | *Se* | Le (a+b+) | 1 | 5 | 24.5 | T>C | 64.5 | 35.5 |
| 235 | *Se* | Le (a+b+) | 1 | 8 | 22 | C | 9 | 91 |
| 244 | *Se* | Le (a+b+) | 1 | 8 | 19.6 | C | 20 | 80 |
| 253 | *Se* | Le (a+b+) | 1 | 22 | 30 | C | 7.5 | 92.5 |
| 259 | *Se* | Le (a+b+) | 1 | 6 | 26.5 | C>T | 42.5 | 57.5 |
| 276 | *Se* | Le (a-b+) | 1 | 7 | 20.7 | C | 15.5 | 84.5 |
| 281 | *Se* | Le (a+b+) | 1 | 6 | 19.9 | C | 3 | 97 |
| 270 | *Se* | Le (a+b+) | 3 | NA | 21 | C | 9.5 | 90.5 |
|  |  |  | 1 | 31 | 33 | C | 7.5 | 92.5 |
| 240^a^ | *Se* | Le (a+b+) | 1 | 9 | 39.6 | C | 95.5 | 4.5 |

Table S1: Secretor profile (*Se*: secretor, *se*: non-secretor) in children (n = 27) who presented F167L RV1 G1P[8] shedding, and information about the immunization schedule (1: 1^st^ dose, 2: 2^nd^ dose, 3: non-vaccinated), number of days of vaccine shedding (NA: not applicable, ? : no information) and rotavirus (RVA) RT-qPCR Ct value. Comparison between results in Sanger Nucleotide Sequencing and Pyrosequencing (Phe: phenylalanine, Leu: leucine) methods in these derived vaccine samples. a. This unique child showed different results in the nucleotide analysis, probably due to these samples having been obtained from the distinctive viral extraction protocols.

| *FUT2* genotyping/ Le (a+b+) secretor phenotype | allele | n (%) |
| --- | --- | --- |
| *Se* ^315C>A^ | heterozygous | 1 (1.3) |
| *Se* ^357T>C^ | heterozygous | 2 (2.6) |
| *Se* ^357T>C^ | homozygous | 3 (3.8) |
| *Se* ^40A>G, 357T>C^ | homozygous | 1 (1.3) |
| *Se* ^(40A>G), 357T>C^ | homozygous | 1 (1.3) |
| *Se* ^40A>G, 357T>C^ | heterozygous | 2 (2.6) |
| *Se* ^357T>C, 480C>T^ | heterozygous | 1 (1.3) |
| *Se* ^357T>C, 481G>A^ | homozygous | 1 (1.3) |
| *Se* ^357T>C, 481G>A^ | heterozygous | 1 (1.3) |
| *Se* ^357T>C, 428G>A, 480C>T^ | heterozygous | 1 (1.3) |
| *Se* ^212T>C, 357T>C, 969C>T^ | homozygous | 1 (1.3) |
| *Se* ^357T>C, 428G>A, 739G>A, (960A>G), (1009A>G), (1011T>C)^ | heterozygous | 1 (1.3) |
| *Se* ^216 C>T, 357T>C, 428G>A, 739G>A, 960A>G, 1009A>G, 1011T>C^ | heterozygous | 1 (1.3) |
| *Se* **^107T>A^**^, 171A>G, 216C>T, 357T>C, 428G>A, 739G>A, 855A>C, 960A>G, 1009A>G,1011T>C^ | heterozygous | 1 (1.3) |
| *Se* ^171A>G, 216C>T, (357T>C), (428G>A), 739G>A, 960A>G, 1009A>G,1011T>C^ | homozygous | 1 (1.3) |
| *Se* ^171A>G, 216 C>T, 357T>C, 428G>A, 739G>A, 960A>G, 1009A>G, 1011T>C^ | heterozygous | 28 (35.9) |
| *Se* ^171A>G, (216 C>T), (357T>C), 428G>A, (739G>A), 960A>G, 1009A>G, 1011T>C^ | heterozygous | 1 (1.3) |
| *Se* ^171A>G, 216 C>T, 357T>C, 428G>A, 739G>A, 1009A>G, 1011T>C^ | heterozygous | 1 (1.3) |
| *Se* ^171A>G, 216 C>T, (357T>C), 375A>G, 428G>A, 739G>A, 960A>G, 1009A>G, 1011T>C^ | heterozygous | 1 (1.3) |
| *Se* ^171A>G, 216C>T, 357T>C, 428G>A, 480C>T, 739G>A, 960A>G, 1009A>G, 1011T>C^ | heterozygous | 2 (2.6) |
| *Se* ^171A>G, 216C>T, (357T>C), 428G>A, 739G>A, 880T>C, 960A>G, 1009A>G, 1011T>C^ | heterozygous | 1 (1.3) |
| *Se* ^171A>G, 216C>T, (357T>C), 428G>A, 739G>A, 960A>G, 1009A>G, 1011T>C^ | heterozygous | 2 (2.6) |
| *Se* ^40A>G, 171A>G, 216C>T, (357T>C), 428G>A, 544G>A; 739G>A, 771G>A, 960A>G, 1009A>G, 1011T>C^ | heterozygous | 1 (1.3) |
| *Se* ^40A>G, 171A>G, 216C>T, 302C>T, 357T>C, 428G>A, 480C>T, 739G>A, 960A>G, 1009A>G, 1011T>C^ | heterozygous | 1 (1.3) |
| *Se* ^40A>G, 171A>G, 216C>T, (357T>C), 428G>A, 739G>A, 960A>G, 1009A>G, 1011T>C^ | heterozygous | 4 (5.1) |
| *Se* ^171A>G, 216C>T, (357 T>C), 428G>A, 481G>A, 739G>A, 960A>G, 1009A>G, 1011T>C^ | heterozygous | 1 (1.3) |
| *Se* ^40A>G, 171A>G, 216C>T,^ *^257C>T^*^, (357T>C), 428G>A, 739G>A, 960A>G, 1009A>G, 1011T>C^ | heterozygous | 1 (1.3) |
| *Se* ^40A>G,^ **^107T>A^**^, 171A>G, 216C>T,^ **^257C>T^**^, (357T>C), 428G>A, 739G>A, 960A>G, 1009A>G, 1011T>C^ | heterozygous | 1 (1.3) |
| without SNPs | - | 14 (17.9) |

Table S2: *FUT2* genotypes in secretors Le (a+b+) children (n = 78 saliva samples), in the birth community-cohort, Rio de Janeiro, Brazil, 2014-2018. Bolded bases are new single nucleotide polymorphisms (SNPs). Bases in parentheses suggest homozygous or heterozygous in relation the genotyping defined in allele column.

| *FUT2* genotyping (non-secretor children) | allele | n (%) |
| --- | --- | --- |
| *se* ^171A>G, 216C>T, 357T>C, 428G>A, 739G>A, 960A>G^ | homozygous | 2 (9.5) |
| *se* ^171A>G, 216C>T, 357T>C, 428G>A, (544G>A), 739G>A, (771G>A), 960A>G^ | homozygous | 1 (4.8) |
| *se* ^171A>G, 216C>T, 357T>C, 428G>A, 739G>A, 960A>G, 1009A>G, 1011T>C^ | homozygous | 14 (58.3) |
| *se* ^171A>G, 216C>T, (315C>T), 357T>C, 428G>A, 739 G>A, 960A>G, 1009A>G, 1011T>C^ | homozygous | 1 (4.8) |
| *se* ^171A>G, 216C>T, (^**^257 C>A^**^), 357T>C, 428G>A, 739 G>A, 960A>G, 1009A>G, 1011T>C^ | homozygous | 1 (4.8) |
| *se* ^171A>G, 216C>T, 357T>C, 428G>A, (542C>T), 739G>A, 960A>G, 1009A>G, 1011T>C^ | homozygous | 1 (4.8) |
| without SNPs | - | 1 (4.8) |

Table S3: *FUT2* genotypes in non-secretor (Le (a+b-) or Le (a-b-)) children (n = 21 saliva samples), in the birth community-cohort, Rio de Janeiro, Brazil, 2014-2018. The bolded base is a new single nucleotide polymorphism (SNP). Bases in parentheses suggest heterozygous.
